# Nanodroplets of Docosahexaenoic Acid-Enriched Algae Oil Encapsulated within Microparticles of Hydrocolloids by Emulsion Electrospraying Assisted by Pressurized Gas

**DOI:** 10.3390/nano10020270

**Published:** 2020-02-06

**Authors:** Cristina Prieto, Jose M. Lagaron

**Affiliations:** Novel Materials and Nanotechnology Group, Institute of Agrochemistry and Food Technology (IATA), Spanish Council for Scientific Research (CSIC). Calle Catedrático Agustín Escardino Benlloch 7, 46980 Paterna, Spain; cprieto@iata.csic.es

**Keywords:** whey protein concentrate, maltodextrin, DHA, algae oil, nanoencapsulation, nutraceuticals

## Abstract

Long chain polyunsaturated omega-3 fatty acids (PUFAs), namely eicosapentaenoic acid (EPA) and docosahexaenoic acid (DHA), are important functional ingredients due to their well-documented health benefits, but highly susceptible to oxidation. One of the most promising approaches to preserve bioactives is their encapsulation within protective matrices. In this paper, an innovative high throughput encapsulation technique termed as emulsion electrospraying assisted by pressurized gas (EAPG) was used to encapsulate at room temperature nanodroplets of algae oil into two food hydrocolloids, whey protein concentrate and maltodextrin. Spherical encapsulating particles with sizes around 5 µm were obtained, where the oil was homogeneously distributed in nanometric cavities with sizes below 300 nm. Peroxide values under 5 meq/kg, demonstrated that the oil did not suffer from oxidation during the encapsulation process carried out at room temperature. An accelerated stability assay against oxidation under strong UV light was performed to check the protective capacity of the different encapsulating materials. While particles made from whey protein concentrate showed good oxidative stability, particles made from maltodextrin were more susceptible to secondary oxidation, as determined by a methodology put forward in this study based on ATR-FTIR spectroscopy. Further organoleptic testing performed with the encapsulates in a model food product, i.e., milk powder, suggested that the lowest organoleptic impact was seen for the encapsulates made from whey protein concentrate. The obtained results demonstrate the potential of the EAPG technology using whey protein concentrate as the encapsulating matrix, for the stabilization of sensitive bioactive compounds.

## 1. Introduction

Long chain polyunsaturated omega-3 fatty acids (PUFAs), namely eicosapentaenoic acid (EPA) and docosahexaenoic acid (DHA), are important functional ingredients due to their well-documented health benefits. At the present time, it is medically proven that long-chain omega-3 PUFAs is related to the growth and development of the brain and retina, heart health, immune-modulating properties as well as protective effects in neurodegenerative diseases [1]. The dietary intake recommendations range from 250 mg per day to 2 g per day according to the Food and Agriculture Organization of the United Nations and World Health Organization [2]. However, the average current intake is far below the recommendations, around 100 mg per day [3]. Hence, the great interest of the food industry in producing omega-3 fortified food products such as mayonnaise, milk, dairy products, bread and bakery, eggs, meat, cereal bars and others [4].

The omega-3 fatty acids, EPA and DHA, cannot be synthesized in the human body, and their supply relies on dietary intake. They are mainly found in marine products such as fish, algae and krill oils [1]. It is really common the use of fish oils as a source of omega-3 fatty acids; however fish oil present certain disadvantages, since fish oil impart their typical fishy flavor when directly added to foods, can be contaminated with methyl mercury, and over-fishing causes a burden on natural fish populations [5]. The use of algae and krill oils, which are the primary source of PUFAs, present the advantage of the absence of pollutants as well as the lack of unpleasant odor [6,7].

Nevertheless, these omega-3 rich oils are highly susceptible to oxidation, involving a loss of nutritional value, the formation of toxic products such as peroxides, and the formation of volatile degradation products that have unpleasant smell and taste, reducing consumer acceptability [8]. For these reasons, the development of efficient strategies is needed to overcome this challenge. One of the most promising approaches to preserve bioactives is its encapsulation within protective matrices, which act as barrier, limiting the contact of the bioactive with the prooxidants in the environment [9]. In addition, encapsulation techniques enhance bioavailability, mask undesirable organoleptic properties and provide a controlled release of these bioactive compounds [10,11]. Spray-drying, freeze-drying, coacervation, fluid bed coating, and hydrogel entrapment are the most commonly used commercial processes for encapsulating omega-3 rich oils until now [12]. However, a wide variety of other methods are in development including spray chilling, extrusion coating, liposome entrapment, and supercritical fluid extraction of emulsions [1,13,14]. Among them, spray drying is the main encapsulation method in the food industry due to its low production costs. However, this technique requires the use of air at high temperatures (170–190 °C), which could contribute to oil oxidation, and produces encapsulates of several microns, which may be too large for certain food applications [13]. A recent review reports very extensively on the latest technologies that have been used to encapsulate omega-3 rich oils [15].

Electrohydrodynamic processing, including both electrospinning and electrospraying, is an emerging technology, which has been used for the encapsulation of hydrophilic and hydrophobic bioactive compounds. In particular, electrospraying is based on the application of a high electric field to a charged polymer solution to produce ultrathin droplets, which after solvent evaporation result in nano- or micro-sized particles. Electrospraying is a straightforward and versatile technique that presents multiple advantages compared to other encapsulation techniques. For instance, electrospraying is carried out at room temperature, which reduces denaturation of bioactives, possesses high encapsulation efficiency, results in decreased particle size and does not require a subsequent step to separate the particles from the medium [9]. Other advantage of the electrospraying process is the wide range of polymeric wall materials that can be used [16].

In the past years, a few studies were reported on the encapsulation of omega-3 rich oils in different materials using the electrohydrodynamic technology [17]. Moomand and Lim encapsulated fish oil into zein fibers by electrospinning [14,18,19]. These authors obtained smooth and thick fibers with a medium size of 500 nm, a theoretical loading capacity of 30% and an encapsulation efficiency over 90%. The fish oil encapsulated into the zein fibers showed higher oxidative stability than the unprotected oil at different storage conditions. Yang et al. encapsulated fish oil into zein-polyvinylpyrrolidone fibers by coaxial electrospinning, obtaining a fiber medium diameter of 560 nm, a loading capacity of 14.5% and an encapsulation efficiency of 97% [20]. García-Moreno et al. used the emulsion electrospinning process to encapsulate cod liver oil into zein, pullulan and poly(vinyl) alcohol (PVA) fibers [21,22,23]. Those biopolymers generate similar results in terms of loading capacity and encapsulation efficiency, around 10% and 90%, respectively; but PVA fibers presented a remarkable lower diameter in comparison to those obtained previously with zein or pullulan (from 600 to 170 nm). However, its oxidative stability was lower due to its high surface area and the prooxidant effect of PVA. Although the electrospinning process is highly promising for food applications, the incorporation of electrospun fibers into food systems could be a challenge due to the high impact on the sensory properties, being its application limited to food products such as cereal bars, granola or biscuits [22]. In this sense, micro and nanoparticles obtained by electrospraying offer a wider range of possibilities. García-Moreno et al. also considered the encapsulation of fish oil into carbohydrate particles by electrospraying, obtaining particles with sizes between 0.1 and 1.5 µm, a theoretical loading capacity of 10% and an encapsulation efficiency around 70%. Nevertheless, these particles showed a poor oxidative stability. Torres-Giner et al. evaluated the encapsulation of fish oil in zein particles by electrospraying obtaining 490 nm particles with a theoretical loading capacity of 33% [10]. These authors stated that the use of zein as shell material allowed to reduce 2.5-fold the oxidation rate constant in comparison with the free oil. Miguel et al. studied the oxidative stability and physical properties of mayonnaise fortified with zein electrosprayed particles loaded with fish oil obtaining an enhanced oxidative stability of the fortified product in comparison with other water soluble electrosprayed encapsulates [24].

In general, one of the main disadvantages of the electrospraying process has typically been its low productivity, normally with a processing throughput of a few milliliters per hour per single emitter [10], which has limited its widespread use for industrial applications. Several technologies have been studied to resolve the scale up issue. Two common approaches to increase throughput were to modify the nozzle design or to increase the number of nozzles. In this context, Fu et al. developed a high-throughput nozzle design, which can increase throughput per nozzle and integrates multiple high-throughput nozzles [25]. Companies like Bionicia S.L. (Paterna, Spain) [26] have developed electrohydrodynamic plants for manufacturing fibers or particles on an industrial scale.

An innovative encapsulation technique based on the combination of electrospraying with the pneumatic atomization process was developed by the research group of Lagaron et al. [5]. This novel high-throughput technology, termed as electrospraying assisted by pressurized gas (EAPG) is based on the atomization of the polymer solution by a pneumatic injector using compressed air that nebulizes within a high electric field. During this process, the solvent is evaporated at room temperature in an evaporation chamber and the encapsulated material is then collected as a free-flowing powder. The potential of this technology was proven for the first time during the encapsulation of omega-3 rich fish oil using zein as a protective encapsulant [5]. However, the disadvantages of zein as an encapsulating matrix are that this maize protein is very expensive and it is not water soluble.

Since the encapsulation efficiency not only depends on the selected encapsulation process but also on the composition of the wall material, in the present work, EAPG technology is applied to encapsulate DHA rich algae oil into two different wall materials that are water soluble and are more cost effective than zein, whey protein concentrate and maltodextrin, to evaluate the influence of the biopolymer on the particle characteristics. Whey protein concentrate was selected due to its good emulsifying and gel-formation properties. They have been claimed to be a good encapsulating agent for oils, fats and volatile compounds, exhibiting effective microencapsulating properties as well as antioxidant properties [27,28]. Maltodextrin is a filler or matrix-forming materials, which is cheap, highly soluble in water and able to form stable emulsions [29]. Therefore, the objective of this work was the comparison of the particle characteristics made from these two biopolymers through the EAPG technology in terms of morphology, encapsulation efficiency, oxidative stability, and organoleptic impact into a model food product.

## 2. Materials and Methods

### 2.1. Materials

Algae oil rich in DHA was supplied by Q’omer Bioactive Ingredients (Valencia, Spain). According to the manufacturer, the algae oil contents DHA in a 40 wt. %. The oil was stored in an airtight container, protected from light at −20 °C. Heat stabilized whey protein concentrate 80% (WPC) was supplied by Davisco Foods International, Inc. (Le Sueur, MN, USA). Whey protein concentrate was claimed to contain 82.4% protein (on dry basis), 5.7% fat, 4.5% moisture, and 4% ash content. The content of lactose was 5.4% lactose (enzymatic assay). Maltodextrin, grade Fibersol-2, was provided by Matsutani Chemical Industry Co. Ltd. (Hyogo, Japan). TEGO SML (sorbitan fatty acid esters) was provided by Evonik Industries (Essen, Germany). Hydrochloric acid (HCl) 37 vol. % was from Sigma Aldrich (St. Louis, MO, USA). Barium chloride dehydrate (BaCl_2_·2H_2_O) (reagent grade), iron (III) chloride hexahydrate (FeCl_3_·6H_2_O) (PRS), chloroform (99%) and methanol (reagent grade) were purchased from Panreac Química SLU (Barcelona, Spain). Iron (II) sulphate heptahydrate (FeSO_4_·7H_2_O) (analytical grade) was from Labkem-Labbox (Mataró, Spain). Ammonium thiocyanate (NH_4_SCN) (99%) and isopropanol (99.5%) were from Acros Organics (Geel, Belgium). 2,2,4-Trimethylpentane (≥99.0%), also known as isooctane, was provided by Honeywell (Morristown, NJ, USA). Ethanol 96 vol. % was purchased from Guinama (La Pobla de Vallbona, Spain). The bottled drinking water was from Agua de Broncales (Teruel, Spain) and skim milk powder from Pirinea (Getafe, Spain). Deionized water was used throughout the study.

### 2.2. Preparation of the Emulsion

Table 1 shows the six different emulsions were prepared to encapsulate the algae oil. Two different algae oil - biopolymer ratios were studied for each biopolymer selected. Aqueous phase of each emulsion was prepared dissolving the biopolymer in the aqueous medium. The organic phase was prepared by dissolving the surfactant in the algae oil. The organic phase was slowly added to the aqueous solution under nitrogen bubbling. The mixture was homogenized with an UltraTurrax T-25 homogenizer (IKA, Staufen, Germany) at 17,000 rpm for 5 min, followed by 5 min of ultrasounds (90%) (Bandelin Sonopuls, Berlin, Germany) under nitrogen bubbling. The emulsion was immersed in a cold water bath in order to avoid temperature increase during homogenization. The emulsion was immediately processed under constant nitrogen bubbling to minimize oil oxidation. Biopolymeric solutions without algae oil were also prepared as the control sample following the same procedure.

### 2.3. Emulsion Droplet Size

The droplet size distribution was measured by laser diffraction in a Mastersizer 2000 (Malvern Instruments, Ltd., Worcestershire, UK). Emulsions were diluted in recirculating water (3000 rpm), until it reached an obscuration of 12%. The refractive indices of sunflower oil (1.469) and water (1.330) were used as particle and dispersant, respectively. Results were given as droplet mean diameter (D_0.5_). Measurements were made in triplicate.

### 2.4. EAPG Process

The emulsion was processed by EAPG using the patented Fluidnatek^TM^ LE500 Capsultek^TM^ pilot plant from Bioinicia S.L. (Valencia, Spain). This proprietary pilot installation by Bioinicia comprises an injection unit, a drying chamber, and a cyclonic collector as described elsewhere [5]. The experiments were optimally performed bubbling continuously nitrogen into the emulsion at controlled ambient conditions, i.e., 25 °C and 30% relative humidity (RH), which was then pumped at 1 mL/min into the injector that worked with an assisted air pressure of 10 L/min and a variable electric voltage from 0 to 30 kV. The generated particles were collected every 20 min from the cyclone and stored in flasks, under vacuum, at −20 °C and protected from light to avoid oxidation.In this particular process, the size of the obtained microparticles depends primarily on the solution properties (mostly solids content and emulsion properties), and process parameters (applied voltage, solution flow rate and air flow rate) [5].

### 2.5. Microscopy

The morphology of particles was analyzed by scanning electron microscopy (SEM) in a Hitachi S-4800 FE-SEM (Hitachi High Technologies Corp., Tokyo, Japan) with an electron beam acceleration of 5 kV. The samples were coated with a gold/palladium layer prior to SEM analysis. Particle diameters were determined using Image J Launcher v1.41 (National Institutes of Health, Bethesda, MD, USA) and the data presented were based on measurements from a minimum of 20 SEM micrographs.The internal morphology of the nanoparticles was studied by transmission electron microscopy (TEM) using a JEOL JEM 1010 (JEOL Ltd., Tokyo, Japan). Sample preparation process consisted of inclusion in LR-white resin and after polymerization, ultrathin section of samples were cut using an ultramicrotome and deposited over the TEM grid [30].

### 2.6. Extractable Oil from the Particles

The quantity of extractable oil from the particles was estimated by measuring the readily soluble algae oil coming out from washing the thin powder with an organic solvent. Thus, quantitative measurements of extractable algae oil were performed by UV-Vis spectrophotometry. For that, 25 mg of small particles were thoroughly washed with isooctane for 30 s and filtered. The absorbance of the filtrate was measured at 285 nm in a UV4000 spectrophotometer (Dinko Instruments, Barcelona, Spain). Standard solutions made of algae oil in isooctane at 0.1–0.5 mg/mL were used to build the standard curve (R^2^ = 0.99), from which the amount of oil present in the filtrate was determined. The percentage of extractable oil was then calculated as follows:

*EO* (%) = (*B*/*A*) × 100% (1)

where *A* is the theoretical amount of algae oil and *B* is the extractable amount of algae oil detected in the filtrate. Measurements were carried out in triplicate. It should be borne in mind that the particles generated by this technology are very small and therefore, extraction by washing with isooctane may remove oil from the surface and near the surface but also from inside the particles.

### 2.7. Peroxide Value Determination

Peroxide value (PV) was used to analyze the oxidative stability of the algae oil. It was determined using the colorimetric ferric-thiocyanate method described by Shantha and Decker [31]. This was based on the principle that lipid peroxides are able to oxidize Fe^2+^ to Fe^3+^ and oxidation can be spectrophotometrically quantified by means of ferric ion complexation with thiocyanate. Peroxide value was determined following the International Dairy Federation standard method ISO3976:1977 [32] with slight modification. Briefly, 0.4 g BaCl_2_·2H_2_O was dissolved in 50 mL of distilled water. Separately, a ferrous solution was prepared by dissolving 0.5 g of FeSO_4_·7H_2_O in 50 mL of distilled water. The barium solution was slowly added to the ferrous one under magnetic stirring, then 2 mL HCl 10 N were added. The BaSO_4_ precipitate was filtered to obtain a clear FeCl_2_ solution, which was stored in an opaque flask. Freshly prepared FeCl_2_ solution was used in each procedure. To prepare the complexing agent, 30 g of NH_4_SCN were dissolved in 100 mL of distilled water.

To determine the peroxide value of the neat algae oil, 8 mg of algae oil were dissolved in 1mL of ethanol 85%. In case of particles, the oil was extracted according to the Bligh and Dyer method [33]. For this, 0.5 or 1 g of sample (in case of 2:1 or 9:1, respectively) were dissolved in 1 mL of deionized water. 0.5 mL of the previous solution were mixed with 1.5 mL of isooctane/isopropanol (2:1 v/v) mixed in the vortex and centrifuged at 1000 rpm for 4 min. The organic phase containing the oil was removed for further analysis.

After that, an aliquot of 200 µL of the oil solutions were mixed with 9.6 mL chloroform-methanol (7:3 v/v). Then, 50 µL FeCl_2_ and 50 µL of NH_4_SCN were added and mixed in the vortex. After 5 min of reaction protected from light, the absorbance was measured at 500 nm against a blank containing all reagents excepting the sample. 

To construct the standard curve of absorbance versus Fe^3+^ concentration, a standard solution of iron (III) chloride was prepared. 0.121 g of FeCl_3_·6H_2_O was dissolved in water and make up to 25 mL. 0.5 mL of the previous solution were made up to 50 mL with chloroform/methanol (7:3 v/v). Standard Fe^3+^ samples containing 0–40 µg Fe^3+^ were analyzed following the previous method by UV-Vis spectrophotometry at 500 nm.

Peroxide value expressed as milliequivalents of peroxides per kilogram of oil, was calculated using the following equation:

*PV* = [(*As* − *Ab*)/*m*] *V*/(2 × 55.84·*m*_0_*S*) (2)

where *As* and *Ab* are the absorbance of the test sample and blank, respectively, *m* is the slope of the calibration curve, *m*_0_ is the weight sample of oil, 55.84 g/mol is the atomic weight of iron, *S* is the volume of the aliquot of the oil solution, *V* is the volume used to dissolve the oil. The samples were measured by triplicate.

### 2.8. Attenuated Total Reflection—Fourier Transform Infrared (ATR-FTIR)

ATR-FTIR spectra of the particles were obtained by using a Bruker Tensor 37 FT-IR Spectrometer (Bruker, Ettlingen, Germany) coupled with the ATR sampling accessory Golden Gate (Specac Ltd., Orpington, UK). Approximately 50 mg of liquid oil and encapsulates were deposited onto the diamond crystal to collect the spectra. All spectra were recorded within the wavenumber range 4000–600 cm^−1^ by averaging 10 scans at 4 cm^−1^ resolution. Measurements were performed in triplicate. Analysis of spectral data was carried out using the OPUS 4.0 data collection software program (Bruker, Ettlingen, Germany).

### 2.9. Stability Tests under Ultraviolet Radiation

An OSRAM Ultra-Vitalux (300 W) lamp (OSRAM, Garching, Germany) was used to accelerate the oxidation of algae oil. This lamp produces a mix of radiation very similar to that of natural sunlight. This blend of radiation is generated by a quartz discharge tube and a tungsten filament. The bulb is made of special glass which allows only that part of the output that is contained in natural sunlight to pass through. The radiation 315–400 nm after 1 h of exposure is of 13.6 W and the radiation between 280 and 315 nm after 1 h of exposure is of 3 W [34,35]. The oxidative stability assay was carried out at ambient temperature under ultraviolet light for up to 10 days. Approximately, 10 g of particles were placed on Petri dishes under an ultraviolet lamp and samples were taken out on daily basis for analysis. Oxidative stability was measured by ATR-FTIR and PV.

### 2.10. Headspace Oxygen Volume Depletion

The oxygen barrier capacity of the different wall materials was studied by measuring the headspace oxygen volume depletion over time at room temperature and 0% RH. For this purpose, a multichannel oxygen meter OXY-4 mini (PreSens Precision Sensing GmbH, Regensburg, Germany) was used. Samples of 2.5 g of sample in case of particles, and the equivalent amount of oil in case of free oil, were placed inside a 100 mL Schleck flask in which 5 mm spot sensors were previously attached. The assays involved the online monitoring of the headspace oxygen using fluorescence decay based on ASTM F2714-08 (2013). Values were taken for 140 h and normalized to the initial oxygen volume. The measurements were done in duplicate. Deviation among different experiments was <2%.

### 2.11. Organoleptic Testing

Organoleptic tests were performed to estimate the impact of adding the particles compared to the neat oil, to reconstituted milk that was used as a food model. The enriched reconstituted milk samples were prepared by adding 37.5 mg of oil or 75 mg of particles to 25 g of skimmed powder milk and 130 mL of bottled drinking water. The organoleptic tests were performed with the freshly prepared particles and after 10 days of accelerated oxidation test under the UV light. Overall fishiness attributes, including taste, odor, and appearance, were evaluated for each sample by six trained panelists from the IATA-CSIC against a reference sample consisting in reconstituted milk without oil or particles. A five point hedonic scale was used to score the samples attributes following next attributes: (0) no difference against reference; (1) little difference against reference; (3) clear difference against reference; (5) big difference against reference.

### 2.12. Statistical Analysis

The results were expressed as mean ± standard deviation. The data were subjected to one-way analysis of variance (ANOVA) using Statgraphics Centurion XVI software (StatPoint Inc., Warrenton, Va, USA). Tukey’s HSD test, at 95% confidence level, was performed to determine the influence of the formulation on the organoleptic impact.

## 3. Results and Discussion

The purpose of this research was to encapsulate algae oil rich in DHA through the EAPG process into two different wall materials in order to compare the particle characteristics in terms of morphology, encapsulation efficiency, oxidative stability and organoleptic impact.

### 3.1. Morphology

**Figure 1:**
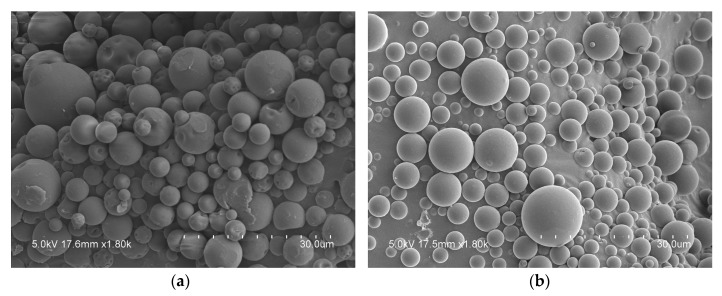
Scanning Electron Microscopy (SEM) images: (**a**) neat whey protein particles; (**b**) neat maltodextrin particles.

SEM images of the neat biopolymer microparticles shown in Figure 1 prove that particles made from whey protein concentrate and maltodextrin are, in general, spherical with a smooth surface, free of cracks, fissures, holes, dents or collapsing, which in principle ensure an adequate protection of the oil. These particles possess a mean particle size of 5.6 ± 2.6 µm and 3.8 ± 1.8 µm, for whey protein concentrate and maltodextrin, respectively. 

**Figure 2:**
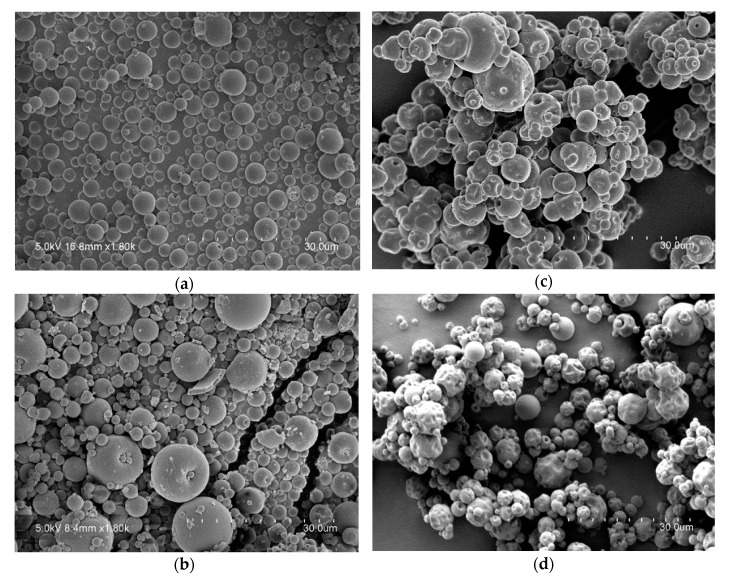
Scanning Electron Microscopy (SEM) images: (**a**) whey protein-algae oil (2:1) particles; (**b**) whey protein-algae oil (9:1) particles; (**c**) maltodextrin-algae oil (2:1) particles; (**d**) maltodextrin-algae oil (9:1) particles.

The incorporation of algae oil into the particles led to a similar morphology for the whey protein concentrate but to a raisin like morphology for the particles made of maltodextrin, especially for the 9:1 ratio composition, as seen from Figure 2. The appearance of dents in oil-loaded maltodextrin samples could be due to a reduced mechanical resistance of the structures in the presence of oil. Table 2 summarizes the particles size for each formulation. Particles with a reduced size are preferred for their incorporation into a food matrix since they might be easier to disperse and could have a lower effect on product sensory properties. On the other hand, a larger surface-to-volume ratio implies an increase of the contact surface between lipids and prooxidants which could negatively affect oxidative stability of the particles [17]. 

### 3.2. Extractable Oil in Isooctane

A thorough oil extraction method was applied to quantify the amount of extractable oil from the particles by UV-Vis spectrophotometry. According to the results presented in Table 2, the extractable oil in organic solvent was lower than 35%. This indicates that a large amount of DHA enriched fish oil, more than 65%, remains strongly bound inside the particles even after exhaustive extraction in an organic solvent. Low oil extraction is considered relevant when working with bioactive compounds prone to oxidation, since it can minimize the exposure of free bioactive compounds to oxygen. These results here are aligned with results obtained previously by Busolo et al., who encapsulated DHA enriched fish oil into zein by this technology, reporting 84% of oil retention within the capsules after organic solvent extraction [5]. García-Moreno et al. reported an even increased oil retention capacity, ranging from 78% to 92% by selecting a different wall material, dextran instead of glucose syrup, using also the EAPG technology [28].

**Figure 3:**
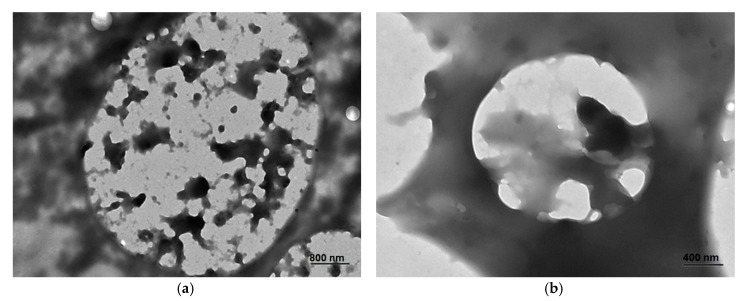
Transmission Electronic Microscopy (TEM) micrographs: (**a**) whey protein concentrate-algae oil particles (2:1), (**b**) maltodextrin-algae oil particles (2:1).

Another important factor affecting the algae oil oxidative stability is its distribution within the particle [18]. According to the TEM micrographs in Figure 3, algae oil was seen to be entrapped blended within the shell material. This kind of internal spongy structure is thought to maximize oil-protection [36]. While in the case of maltodextrin, the oil seems dispersed inside the particle into pockets with an average drop size of 300 nm (Figure 3b); in the case of whey protein concentrate, the oil was seen more homogenously distributed in cavities with an average size of 200 nm (Figure 3a). The size of these nanocavities is in good agreement with the size of the droplets in the emulsion gathered also in Table 2. The observed morphological difference could imply a more efficient protection of the lipid molecules by the protein, also suggested by the lower PV values (see the discussion below) gathered in Table 2. From the morphology observations, it seems that the oil droplets are trapped inside the particles and not outside or at the surface, but some degree of porosity at the particles surface may explain that some of the oil may be extractable in contact with an organic solvent that solubilizes the oil.

### 3.3. Oxidative Stability

The protective effect of the different encapsulating materials on the oxidative stability of algae oil was assessed through an accelerated degradation assay under ultraviolet (UV) light in comparison with the oxidative stability of the free oil during 10 days. For this purpose, the peroxide value and the relative intensity of characteristic infrared bands of the algae oil were measured during the assays. 

The peroxide index serves to quantify the primary oxidation products of the fatty acids, concretely the hydroperoxides. As it can be observed in Table 2, most of the samples present an initial peroxide value after encapsulation under 5 meq/kg, which is in concordance with the Global Organization for EPA and DHA omega-3s (GOED) [37]. Being the initial value of PV of the algae oil of 1.6 ± 0.9 meq/kg. The low peroxide value obtained for the encapsulates confirms that the encapsulation process was performed under mild conditions not affecting the oil composition. It can be considered that, even though the encapsulation process was carried out using air flow at room temperature, oil oxidation was limited by the continuous bubbling of nitrogen to the emulsion during the process as well as to the frequent withdrawal of the product from the collector and subsequent storage under vacuum. The little difference between the peroxide value of the pure fresh oil and the encapsulates might be attributed to the lipid oxidation during the emulsion preparation due to oxygen inclusion, to the increase in specific area surface, and to the encapsulation process as a result of the exposure of the surface oil to atmospheric air during production [28].

**Figure 4:**
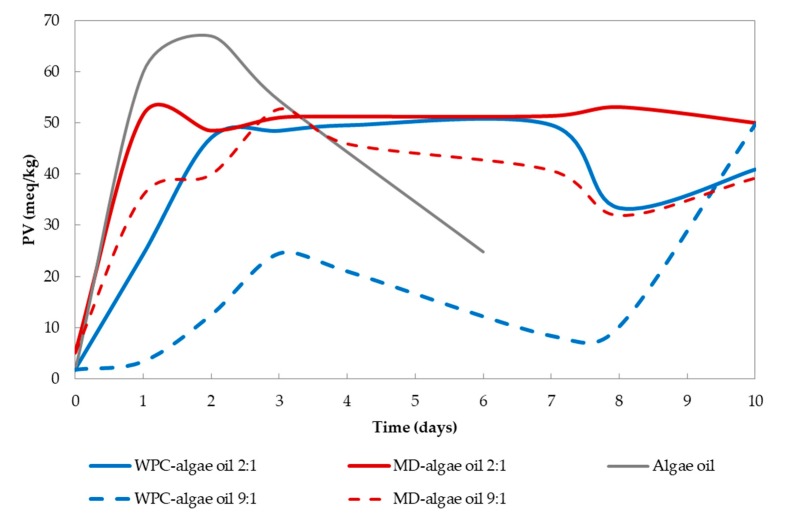
Evolution of the peroxide value in the particles encapsulating algae oil in whey protein concentrate and maltodextrin in comparison with the free oil. Line represent ratio 2:1, while dashed line represents ratio 9:1.

Regarding the oxidative stability when exposed to UV light, Figure 4 indicates that PV increased rapidly in all samples between days 0 and 3 because of the primary oxidation of the PUFAs within the algae oil. Although hydroperoxides are tasteless, they decompose rapidly into secondary oxidation products (presumably aldehydes, ketones, and alcohols of distinct chain lengths and degrees of saturation) in the presence of heat or metal ions, which are responsible for the off-flavours [21,38], and as a consequence, the PV decreases after the initial rising. According to Figure 4, the beginning of the secondary oxidation takes place between days 1 and 2 for particles containing 33% of algae oil, whereas for the particles containing 10% of oil, secondary oxidation reactions began after the third day. In samples containing whey protein concentrate the increase and also the decrease in the PV was less accused compared to the polysaccharide, demonstrating the inherent antioxidant properties of these proteins provided by the lactoferrin chelating capacity of transition metals and the free radical scavenging of the amino acids containing sulfhydryl groups (e.g., cysteine) [39]. In the neat algae oil, the hydroperoxides concentration was significantly higher than that of the encapsulated oil, and after the second day, the secondary oxidation reactions began. It was not possible to measure PV after the sixth day in the pristine oil due to gelation under the UV light.

The PV method anticipates and quantifies the primary oxidation products and also gives information regarding the indirect presence of other species as a consequence of the secondary oxidation reactions. The off-flavors formed from omega-3 PUFAs secondary oxidation reactions are particularly unpleasant and the sensory threshold for many of these oxidation products is really low [38]. So it is in fact the secondary oxidation what is a major concern for polyunsaturated oils. 

**Figure 5:**
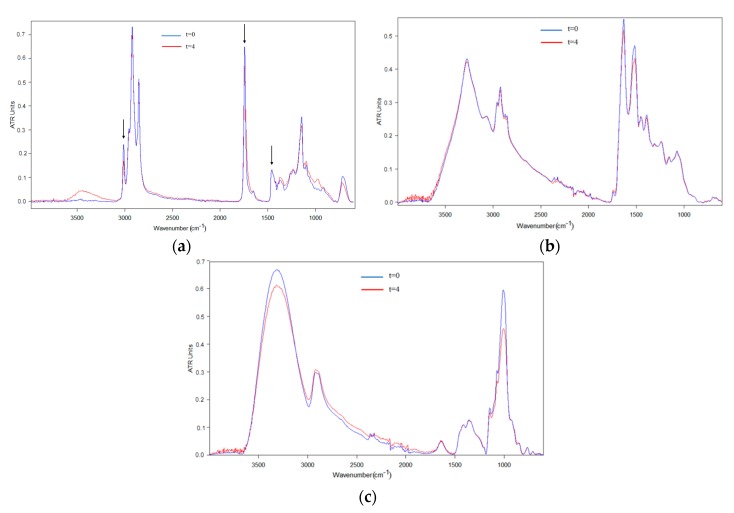
Comparison of raw materials before and after being exposed to 4 days of UV light. (**a**) Free algae oil; (**b**) Whey protein concentrate; (**c**) Maltodextrin. The spectra were normalized, for comparative purposes, to the intensity of the algae oil internal standard band at ca. 1456 cm^−1^.

**Figure 6:**
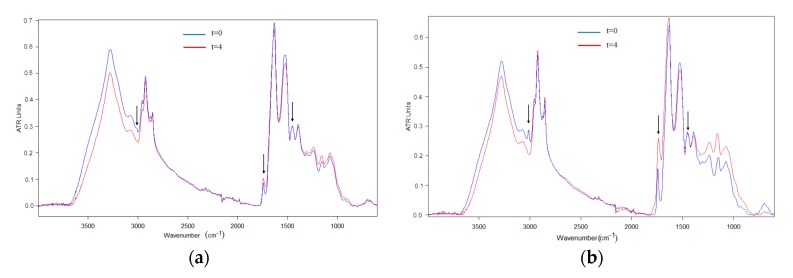
Comparison of the algae oil-loaded WPC particles spectra before and after 4 days of UV exposure. (**a**) WPC-algae oil 9:1; (**b**) WPC-algae oil 2:1. The spectra were normalized to the intensity of the internal standard band of the algae oil at ca. 1456 cm^−1^.

**Figure 7:**
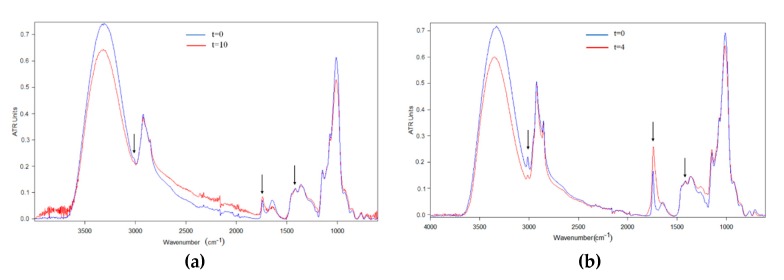
Comparison of the algae oil-loaded MD particles spectra before and after 4 days of UV exposure. (**a**) MD-algae oil 9:1; (**b**) MD-algae oil 2:1. The spectra were normalized to the intensity of the internal standard band of the algae oil at ca. 1456 cm^−1^.

In order to put forward alternative more comprehensive methods to those already proposed in the literature, the chemical changes in the algae oil were also followed by ATR-FTIR spectroscopy. FTIR spectroscopy has already proven to be an easy and reliable method to ascertain the stability of microparticles encapsulating fish oil prepared by spray drying [40]. First, the ATR-FTIR spectra of the free oil during the oxidation process were studied. Figure 5a shows the comparison of the normalized in intensity ATR-FTIR spectrum of the fresh free algae oil with the normalized in intensity ATR-FTIR spectra for the free oxidized algae oil after 4 days under UV light exposure. The reference band used as internal standard (see arrows to the right in Figure 5, Figure 6 and Figure 7) for normalization was the band at ca. 1456 cm^−1^, which is assigned to rocking vibrations of (C–H) bonds of cis-disubstituted alkenes. This band was used as an internal reference to follow oxidation, because this band did not show any variation during oxidation, as observed during the ATR-FTIR monitoring of the in situ oxidation of a drop of oil deposited on the ATR crystal (results not shown). Similar results were obtained by Guillén and Cabo when studying the oxidation of edible oils using FTIR [41]. The characteristic bands of the algae oil were clearly visible in the ATR-FTIR spectra, as shown in Figure 5a. The band at 3012 cm^−1^ (see arrow to the left in Figure 5, Figure 6 and Figure 7) corresponds to the stretching of cis-alkene groups –HC=CH– in PUFAs, whose intensity decreased as oxidation progressed, as a consequence of the disappearance of the unsaturations [18,42]. Another characteristic band of the oil is at ca. 1741 cm^−1^ (see central arrow in Figure 5, Figure 6 and Figure 7) which is assigned to the C=O stretching of ester and acid groups in triglycerides [18,43]. This band suffered a decrease in intensity and widened towards lower wavenumbers as oil oxidation progressed. This was due to the generation of hydroperoxides, aldehydes, ketones and alcohols, as well as other molecules related to secondary oxidation [41]. Other bands at ca. 1238 and 1163 cm^−1^ that could also be clearly seen have been ascribed to the proportion of saturated acyl groups in the sample. The position of both bands shifted towards higher wavenumbers during the oxidation process, which is indicative of the formation of smaller saturated acyl molecules as a result of fatty acid degradation [41]. Furthermore, a relative increase in absorbance has been noted in the region at ca. 971 cm^−1^, assigned to trans-double bonds, indicating the increase in this type of bond as oxidation progresses [43]. Finally, the band at ca. 705 cm^−1^, attributed to the overlapping of the methylene rocking vibration and the out-of-plane bending vibration of cis-disubstituted olefins, showed an increase in frequency as oxidation products were formed [41].

Figure 5b,c show, as an example, the spectra of the raw materials, whey protein concentrate and maltodextrin respectively, before and after 4 days of UV light exposure. Those materials did not significantly alter their spectra as a result of UV light exposure, beyond some reduction in the typical water bands, most likely associated to the heat generated by the UV lamp. 

The ATR-FTIR spectra of the algae oil-loaded encapsulated particles were also studied over time during exposure to UV light. Figure 6 and Figure 7 summarize the relative spectral changes of the characteristic bands of the algae oil for the samples of whey protein concentrate and maltodextrin respectively. Comparing the spectra of the particles with those of the raw materials, it is possible to conclude that the main changes in the characteristic bands are due to the oxidation of the oil, which becomes more significant for the capsules with higher oil content. 

**Figure 8:**
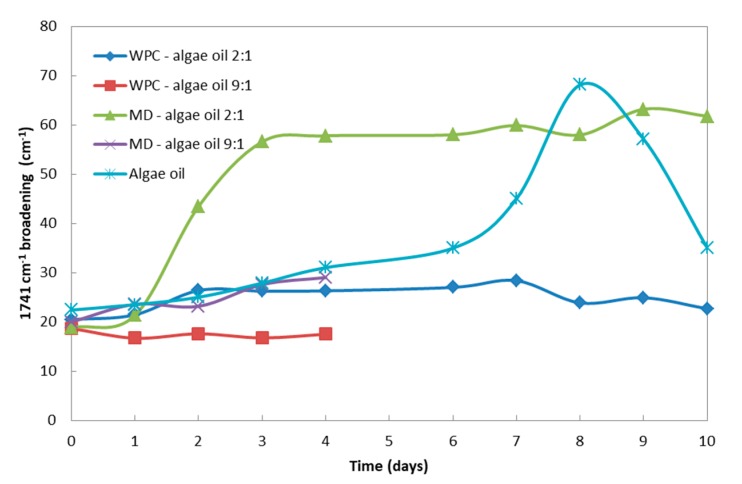
Peak broadening of the samples, measured as the 1741 cm^−1^ band width at half height, as a function of UV light exposure for up to 10 days.

After careful examination and characterization of the different changing features in the spectra of the purposely oxidized encapsulates, it was decided that the safer parameter to be used to assess the oxidative effect, particularly in regard to secondary oxidation, was the band broadening of the carbonyl band at ca. 1741 cm^−1^. The reason for this selection is that changes in relative intensities in complex composite materials become more difficult to assign in an unambiguous manner. Thus, Figure 8 shows our proposal for a simple method to assess secondary oxidation by following the band width at half height of the 1741 cm^−1^ band, measured over time upon UV light exposure. From observation of this figure, it is possible to conclude that in the case of the free algae oil, the band broadening was seen the most intense in the first day, and increased dramatically after the sixth day, as a consequence of the generation of more secondary oxidation products, that may create higher organoleptic impact. The sample of MD-algae oil 2:1 suffered a significant broadening after the first day, suggesting the strongest secondary oxidation of all encapsulates. On the other hand, the sample with whey protein concentrate did not show a significant broadening during the time lapse studied, and neither did the samples with biopolymer: algae oil ratios of 9:1, most likely due to the significantly lower amount of oil in the sample. In any case, by looking at the samples with a 9:1 ratio, only evaluated during the first four days, it is again observed that the MD matrix seems to be less efficient at blocking secondary oxidation. As a consequence of the observations related to PV and ATR-FTIR experiments, the protein seems to block or reduce the extension of the secondary oxidation reactions by blocking more efficiently UV light [44,45] and serving as gas and organic vapor barrier material at ambient conditions, compared to the polysaccharide.

### 3.4. Headspace Oxygen Depletion 

**Figure 9:**
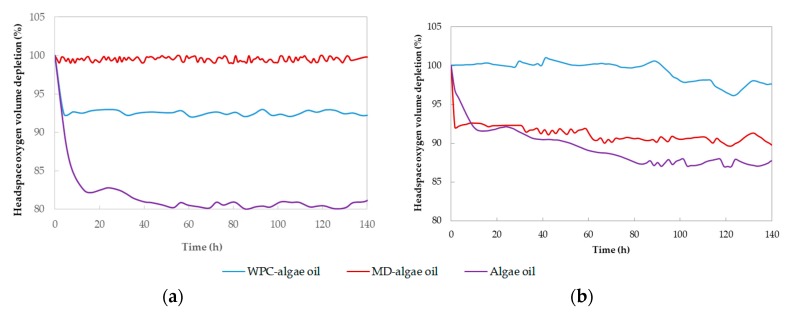
Averaged evolution of the percentage of headspace oxygen volume depletion over time for the free and encapsulated algae oil in whey protein and maltodextrin particles; (**a**) polymer:oil of 2:1 ratios, (**b**) polymer:oil of 9:1 ratios.

This analysis was done to assess the oxygen barrier effect brought forth by the encapsulating materials, which importance on delaying lipid autooxidation was stated on a previous study encapsulating fish oil by spray drying [46]. Figure 9 shows the percentage of headspace oxygen volume depletion, presumed consumed by oil oxidation, asdetermined by the fluorescence decay method, for an equivalent amount of algae oil, in free and encapsulated samples. In the encapsulated sample, the signal of the matrix, in any case not very relevant, was subtracted in order to show and compare just the oxygen consumption by the oil. The study was performed at room temperature 25 °C and 0% RH. This technique has been used before to determine oxygen permeability and scavenging in sealed packaging materials [47]. However, in the present study it was applied to monitor the oxidation of algae oil in order to assess the efficiency against oxygen permeation provided by the encapsulation technologies used. As it can be seen from observation of Figure 9, free liquid algae oil oxidized significantly faster before arresting the trend during testing, than the one encapsulated in the microparticles. The initial rapid oxidation rate observed, may be related to the oxidation of the available surface of the liquid oil and of the oil in the surface layers of the solid particles. After 100 h of experiment and at 0% RH, the free oil consumed around 20% of the oxygen present, whereas the particles with a ratio polymer:oil of 2:1 consumed ca. 7% of oxygen at its maximum, corresponding to the protein (Figure 9a). It should be born in mind that the particles spread over the testing glass surface are expected to have more area of exposition to oxygen, due to surface roughness and higher relative mass, than the liquid oil. Interesting, the oil encapsulated in the MD, was seen to consume very little oxygen during the time interval studied, suggesting that for this polymer:oil composition, the morphology of the protein can comparatively still be improved to reduce availability of oil for oxidation. On the other hand, and under same testing conditions, for the particles with 9:1 ratios (see Figure 9b), the free liquid oil consumed around 12% of the oxygen, whereas the fine encapsulates consumed 10% at its maximum. In this case, whey protein was the one not seen to consume almost any oxygen, compared to the MD. Whey protein concentrate is known to provide an excellent barrier to oxygen permeation, especially at dry conditions [48]. Maltodextrin has also demonstrated to exhibit a good barrier to oxygen [49]. The reason for the different behavior in regard to the different consumption of oxygen depending on the material and polymer:oil ratios, may be related to the attained capsule morphology (see discussions in relation to Figure 2). In addition, Figure 4 already indicated that the PV evolution showed more similar performance for the MD capsules regardless of oil concentration, suggesting that reducing oil concentration was less efficient at avoiding oxidation for MD than it definitely is for the protein.

### 3.5. Organoleptic Properties

Finally, an organoleptic test was performed to assess the organoleptic impact of the encapsulates in comparison with the free oil, using reconstituted milk powder as a model food product. This test was performed with the freshly prepared encapsulates and with encapsulates after 10 days of accelerated oxidation test under UV light. 

**Figure 10:**
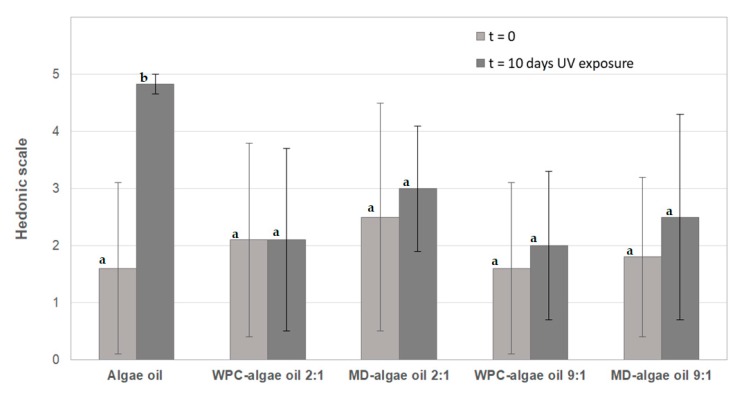
Comparison of the sensory panelists score of reconstituted powdered milk samples containing freshly prepared free and encapsulated algae oil particles at time cero and after 10 days of UV light exposure. Data are represented as mean ± standard deviation. Different letters indicate significant difference among samples (*p* < 0.05).

According to Figure 10, trained panelists found little difference, not significant, between the fortified milk containing the encapsulates or the free algae oil at time cero. This is due to the known reduced organoleptic impact of the algae oil, and confirms that the oil was not degraded during the encapsulation process, due to the mild processing conditions at room temperature utilized by the EAPG encapsulation method. Whey protein concentrate tends to provide the lowest organoleptic impact trend in comparison with maltodextrin, albeit differences are not statistically significant. The organoleptic impact also tended to be reduced by increasing the ratio polymer:algae oil. However, after 10 days of accelerated oxidation test, the oil showed a strong organoleptic impact, whereas the organoleptic impact in the encapsulates was not significantly affected, always with a better performing trend for the protein, in agreement with the above mentioned PV and ATR-FTIR results.

## 4. Conclusions

Industry is highly interested in the encapsulation of bioactive compounds to be incorporated in food, pharmaceutical and cosmetics products, and has demonstrated a special interest in the encapsulation of omega-3 polyunsaturated fatty acids due to their claimed health benefits. However, their high susceptibility to oxidation brings about a huge challenge. Up to now, a wide variety of encapsulation technologies have been developed, but most of them result in oxidation to a higher or lower degree of the bioactive or in scaling difficulties. In this work, the innovative high-throughput emulsion EAPG technique was successfully employed to encapsulate algae oil in two different food matrices, whey protein concentrate and maltodextrin, being the extractable oil in organic solvents lower than the 35%, and minimizing oil oxidation due to processing at room temperature and the fast evaporation characteristics of the EAPG process. Spherical particles with sizes around 5 µm were obtained, with the oil being homogeneously distributed in nanometric cavities. Best results in terms of oxidative stability were achieved with whey protein, which provided better protection to UV light and oxygen permeation, especially at low oil loading ratios. According to the ATR-FTIR spectroscopy new methodology developed, the protein could reduce the extension of the oil secondary oxidation reactions in comparison with the free oil and the oil encapsulated in the polysaccharide. Finally, the organoleptic impact of the produced particles was studied in a model food product, i.e., reconstituted powdered milk. Trained panelists found little difference between the fortified food product and the reference, in particular for the whey protein encapsulates, as a consequence of the oxidative stability procured by the developed encapsulation process. The obtained results suggest that the EAPG process could become a very promising technique for the microencapsulation of sensitive materials, such as nutraceuticals, which can be used thereafter to develop functional food products, pharmaceuticals or cosmetics.

## Figures and Tables

**Table 1:** Formulation of the emulsion used to encapsulate algae oil. W means aqueous phase, O organic phase.

Formulation	[Biopolymer]	[Surfactant]	Ratio	Ratio
	(%wt.)	(%wt.)	O:W	biopolymer:algae oil
WPC - algae oil 2:1	22.5	9.1	11.0:89.0	2:1
WPC - algae oil 9:1	22.5	31.0	3.5:96.5	9:1
MD - algae oil 2:1	22.5	9.1	11.0:89.0	2:1
MD - algae oil 9:1	22.5	31.0	3.5:96.5	9:1

**Table 2:** Characteristics of the various encapsulates. D (0.5), mean droplet size; EO, extractable oil; PV, peroxide value.

Formulation	Emulsion Droplet Size D (0.5) (µm)	Capsule Size (µm)	EO (%)	PV (meq/kg)
WPC-algae oil 2:1	0.207 ± 0.003	2.8 ± 0.9	35 ± 5	1.9 ± 0.4
MD-algae oil 2:1	0.272 ± 0.002	4.9 ± 1.3	15 ± 1	5.1 ± 2.8
WPC-algae oil 9:1	0.184 ± 0.002	3.0 ± 2.4	21 ± 2	3.0 ± 2.5
MD-algae oil 9:1	0.173 ± 0.001	3.4 ± 2.2	23 ± 9	4.8 ± 0.7
